# Inquiry Into Physicians’ Scope of Practice in Japanese Rural Hospitals During the COVID-19 Pandemic: A Serial Cross-Sectional Study

**DOI:** 10.7759/cureus.26164

**Published:** 2022-06-21

**Authors:** Masayoshi Kusunoki, Ryuichi Ohta, Kentaro Suzuki, Takayuki Maki, Chiaki Sano

**Affiliations:** 1 Emergency Medicine, Shimane Prefectural Central Hospital, Izumo, JPN; 2 Community Care, Unnan City Hospital, Unnan, JPN; 3 Cardiology, Matsue Seikyo General Hospital, Matsue, JPN; 4 Community Medicine Management, Shimane University Faculty of Medicine, Izumo, JPN

**Keywords:** inquiry, pandemics, covid-19, japan, urban population, rural population, scope of practice

## Abstract

Introduction

Physicians' scope of practice (SoP) depends on clinical settings and is related to how motivated they feel. The clarification and differences in the SoP in each clinical setting are necessary for physicians’ careers. This study aimed to investigate how coronavirus disease 2019 (COVID-19) affected physicians’ SoP.

Methods

This serial cross-sectional study compares the differences in physicians’ SoP among Japanese rural community hospitals between 2018 and 2020. The participants were admitted patients in the internal medicine wards of the two community hospitals in urban and rural districts in the rural prefecture (Shimane) of Japan from January 1, 2018, to December 31, 2020. We calculated the number of health problems among the highest 50% of all health problems for each physician (SoP-50%) and used it as an indicator of the comprehensiveness of clinical practice.

Results

The study found that SoP-50% was significantly higher in rural districts in 2018 (p = 0.0209). This trend remained unchanged even during the COVID-19 in 2020 (p = 0.0441). While there was a significant regional difference in the SoP, pre and post-COVID-19 analysis of the SoP in each region did not show any significant change.

Conclusion

This is the first study to indicate that greater comprehensiveness of clinical practice is required in the districts of rural Japan. The findings can be helpful for physicians’ medical education and career choices.

## Introduction

The scope of practice (SoP) can be beneficial in describing the characteristics of physicians and clinical settings. The SoP refers to physicians’ comprehensiveness in primary care settings, wherein a strong foundation is required for medical care. Donaldson (1994) defined primary care as “the provision of integrated, accessible health care services by clinicians who are accountable for addressing a large majority of personal health care needs, developing a sustained partnership with patients, and practicing in the context of family and community” [1]. This statement implies that one of the characteristics of primary care providers is their broad SoP and that it covers various concerns overarching different medical categories.

The range of the SoP varies in the training of physicians and the working environment. For instance, the clinical competencies required from rural physicians differ from those of urban physicians. A study conducted in Canada identified a variation in clinical activity across different practice locations. Generally, rural physicians tend to work more hours and engage in a broader spectrum of clinical activities, which indicates that the SoP is driven by patient and community needs [2]. Another study suggested that the SoP of rural physicians is broad in two aspects: the categories of age, such as from infants to the elderly, and the categories of care, such as primary healthcare, primary prevention, emergency care, and end-of-life care [3]. These results imply that a broad SoP is crucial, especially in remote areas where medical resources are limited.

Patient characteristics are another critical factor that affects the SoP of physicians. The demand for primary care physicians with a comprehensive SoP is exceptionally high in an environment where there are many elderly patients with multimorbidity. Japan is known as one of the countries at the forefront of the “Super-Aging Society.” It has the world’s highest proportion of people aged over 65 years, which was 28.4% of the country’s population in 2019 [4]. One of the inevitable problems of an aging society is the tremendous increase in healthcare costs. According to the national statistical survey conducted by the Ministry of Health, Labor and Welfare, more than 70% of healthcare recipients in Japan consist of individuals over 65 years of age [5]. Currently, older patients are taken care of by various medical professionals, described as “polydoctors,” further increasing the cost of care. The polydoctor situation can cause a polypharmacy concern because of the prescription cascade, causing more symptoms in older patients. In countries like Japan, with many elderly patients, physicians with a comprehensive SoP play a crucial role in achieving better healthcare outcomes by managing multiple health problems. The numerous benefits of these physicians include an increase in accessibility, coordination, and accountability for the individual [6]. Furthermore, a wider SoP is associated with greater equity, improved continuity, and better health outcomes at lower costs [7]. Therefore, it is critical to secure enough physicians with clinical comprehensiveness in regions populated by a majority of elderly who have multiple comorbidities.

Well-established educational programs are essential for imparting clinical comprehensiveness to a general physician [8]. One of the ideal environments for medical students and residents to acquire the necessary knowledge and skills to become competent general physicians is community medical institutes, where the SoP is broader than that of tertiary institutes. Hands-on training in community hospitals can be an effective tool for medical students to learn and expand their knowledge about general medicine [9]. The educational program for medical students in Japan is predominantly designed by medical professors who are organ-specific specialists and, therefore, they lack the perspective of general physicians. Elucidating the role of general physicians in community hospitals may enhance the establishment of educational programs that provide more exposure to such clinical settings. Additionally, the clarification of physicians’ SoP in the particular working environment will be beneficial to medical students and physicians in their career selection and the recruitment of physicians. However, few studies have been conducted on the differences in physicians’ SoP in rural and urban districts in Japan. One of the primary purposes of this study was to compare the geographical variance of physicians’ SoP in the same prefecture in Japan.

Another focus of this study is to elucidate the change in physicians’ SoP during the coronavirus disease 2019 (COVID-19) pandemic. The pandemic has undoubtedly affected many aspects of medical care in a number of ways. A study showed an association between the COVID-19 pandemic and the proportion of comprehensiveness of medical care among rural patients [10]. However, there are no studies investigating the effect of the COVID-19 pandemic on physicians’ SoP. We investigate how the physicians’ SoP changed through the COVID-19 pandemic and its differences among rural and urban regions in the same prefecture in Japan.

## Materials and methods

This serial cross-sectional study compares the differences in physicians’ SoP among Japanese rural community hospitals between 2018 and 2020. The participants were admitted patients in the internal medicine wards of the two community hospitals in urban and rural districts in a rural prefecture (Shimane) of Japan from January 1, 2018, to December 31, 2020.

Setting

Shimane (6,708 km2) is a rural prefecture in western Japan. In December 2020, the total population was 666,211 (321,846 males and 344,365 females); 34% of the population was over 65 years of age. The prevalence of COVID-19 in Shimane was 0.032% (214/666,211) in 2020 [10].

Matsue (572.99 km^2^) is the most urbanized city in Shimane. In March 2020, the total population was 202,991 (97,898 males and 105,093 females); 30.5% of the population was over 65 years of age. Matsue has 180 clinics, 41 homecare stations, 31 visiting nurse stations, two public hospitals, and nine private hospitals. The number of doctors per 100,000 population in Matsue was 271.54 (November 2018). Matsue Seikyo General Hospital is a private community hospital in the city, with 351 care beds, 20 physicians working as internists, and 213 nurses in 2020. The COVID-19 prevalence in Matsue was 0.075% (153/202,991) in 2020 [10].

Unnan (553.4 km2) is a remote rural city in Shimane. In March 2020, the total population was 37,637 (18,145 men and 19,492 women); 39% were over 65 years of age. The city has 16 clinics, 12 homecare stations, three visiting nurse stations, and one public hospital. The number of doctors per 100,000 population in Unnan was 138.29 (November 2018). In 2020, Unnan City Hospital had 281 care beds, 12 physicians working as internists, and 197 nurses. The COVID-19 prevalence in Unnan was 0.0079% (3/37,637) in 2020 [10].

Participants

The participants of this study were all the patients admitted to the internal medicine wards in two community hospitals, representing a rural and an urban region, from January 1, 2018, to December 31, 2020. The two hospitals were selected because they had a similar number of beds and catered to similar population sizes under usual circumstances.

Measurement

Patient information regarding their age, sex, and diagnosis at the time of admission were obtained from the electronic medical records of the two hospitals. We classified the diagnoses of all patients using the International Classification of Diseases, Tenth Revision (ICD-10), which was used in the two hospitals during this period. This study focused primarily on the difference in physicians’ SoP in urban and rural community hospitals. To objectively assess this difference, we calculated the number of types of health concerns constituting the higher 50% of all health problems seen by each physician (SoP-50%), an indicator of the comprehensiveness of clinical practice [11].

Data analysis

We analyzed the patient characteristics of the two hospitals according to two variables: sex and age. First, we calculated the SoP-50% of each physician and compared the value between the two hospitals in 2018 and 2020. The data were compared based on the period (pre or post-COVID-19) and the regional aspect (urban or rural). Since the sample size (number of physicians) was not extensive enough to assess the standard normal distribution pattern of the data, we used the Mann-Whitney test for non-parametric data. Stata 16 (StataCorp. 2019. Stata Statistical Software: Release 16) was used for statistical data analysis. In the present study, the appropriate sample size of the number of physicians was calculated based on the data of physicians’ SoP in 2020, with a significant level of 0.05 and a power of 0.8. A sample size of 17 was determined to be sufficient to detect the significant differences in SoP.

Ethical considerations

This study was approved by the Matsue Seikyo General Hospital Ethics Committee (approval code: 120401). All the personal information of the participants was anonymized when it was extracted from the electronic medical records; hence, it was not possible to identify a person from the dataset.

## Results

Study setting

The demographic features of the patients admitted to the internal medicine wards in each hospital in 2018 and 2020 are presented in Table 1. There was a notable decrease in the number of admitted patients in the urban group (n=2,142 in 2018, n=1,776 in 2020), whereas it was relatively the same in the rural group (n=1,075 in 2018, n=1,023 in 2020). In the urban group, the mean age in 2020 was significantly higher than that in 2018 (p < 0.001). Conversely, in the rural group, the mean age in 2020 was significantly lower than that in 2018 (p < 0.001). In terms of sex distribution, there was no significant difference in both urban and rural groups.

**Table 1:** Demographic features of the admitted patients in the two hospitals in 2018 and 2020

	Urban (Matsue)							Rural (Unnan)				
Year	2018 (n = 2,142)			2020 (n = 1,776)		P-value	2018 (n = 1,075)			2020 (n = 1,023)		P-value
Female, n, %	1,062	49.58	897		50.51	0.563	564	52.47	525		51.32	0.6
Male, n, %	1,080	50.42	879		49.49		511	47.53	498		48.68	
Mean age (SD)	77.27 (14.29)			78.79(13.86)		<0.001	82.63 (15.51)			79.43 (16.54)		<0.001

Health problems

The 15 predominant health problems in the two hospitals in 2018 are listed in Table 2. In 2018, “Pneumonitis due to inhalation of food and vomit” (5.8%) was the highest in the urban group while it was “Polyp of the colon” (5.7%) in the rural group. Infectious diseases contributed significantly to complete disease distribution in both groups. The total proportion of infectious diseases in the 15 predominant health problems was slightly higher in the rural group (20.5%) than the urban group (16.1%). In the urban group, cardiovascular diseases, such as “Atrial fibrillation,” “Heart failure,” “Supraventricular tachycardia,” and “Angina,” constituted a significant section of the disease distribution (17.8%). In contrast, the total cardiovascular disease percentage in the rural group was 4.5%, including “Acute heart failure” and “Congestive heart failure.”

**Table 2:** Top 15 health problems in the two hospitals in 2018 *The denominator of proportion is the total number of patients admitted to internal medicine wards in each hospital.

Urban (Matsue)					Rural (Unnan)				
Rank	Health problem	Proportion*		ICD-10 code	Rank	Health problem	Proportion*		ICD-10 code
1	Pneumonitis due to inhalation of food and vomit		5.8%	J690	1	Polyp of colon		5.7%	K635
2	Congestive heart failure		5.5%	I500	2	Urinary tract infection		5.5%	N390
3	Other forms of angina pectoris		5.4%	I208	3	Unspecified bacterial pneumonia		5.3%	J159
4	Urinary tract infection		4.6%	N390	4	Sepsis		3.7%	A419
5	Paroxysmal atrial fibrillation		4.0%	I480	5	Dehydration		3.0%	E86
6	Pneumonia, unspecified organism		3.5%	J189	6	Acute heart failure		3.0%	I509
7	Cerebral infarction due to embolism of cerebral arteries		2.8%	I634	7	Pneumonia, unspecified organism		2.8%	J189
8	Dehydration		1.8%	E86	8	Pneumonitis due to inhalation of food and vomit		2.3%	J690
9	Persistent atrial fibrillation		1.7%	I481	9	Congestive heart failure		1.5%	I500
10	Polyp of the colon		1.5%	K635	10	Cerebral infarction due to thrombosis of cerebral arteries		1.4%	I633
11	Diverticular disease of large intestine		1.4%	K573	11	Acute respiratory failure		1.0%	J9609
12	Supraventricular tachycardia		1.2%	I471	12	Infectious gastroenteritis and colitis, unspecified		0.9%	A099
13	Encounter for observation for other suspected diseases and conditions ruled out		1.2%	Z038	13	Type 2 diabetes mellitus		0.9%	E11
14	Sepsis		1.1%	A419	14	Nutritional deficiency, unspecified		0.9%	E639
15	Calculus of bile duct with cholangitis		1.1%	K803	15	Other peripheral vertigo		0.9%	H813

Table 3 shows the 15 predominant health problems in the two hospitals in 2020. Some of the trends in disease distribution were unchanged from 2018. In urban and rural areas, infectious diseases, such as urinary tract infections and pneumonia, constituted a significant section of the distribution. The total proportion of infectious diseases in the 15 predominant health problems was slightly higher in the rural group (22.3%) than the urban group (18.9%), similar to the case in 2018. Another common health problem is heart failure. Heart failure was subdivided into “Congestive heart failure” and “Acute heart failure” in both groups. Combining these two diagnostic titles, the proportion of patients with heart failure was higher in the urban group (8.0%) than in the rural group (5.5%), both of which constitute a significant proportion of the entire distribution. Some of the notable differences in the health problem distribution between the two groups include the significantly larger proportion of “Polyp of the colon” admissions in the rural group (6.4% in the rural and 1.6% in the urban group) and “Atrial fibrillation” admissions (3.7%; “Persistent” and “Paroxysmal” combined in the urban, and 0% in the rural group). While most of the tendencies were unchanged from 2018, there are some notable differences. In 2020, COVID-19 held the 11th position (1.8%) in the rural group, whereas there were no admissions under this diagnosis in the urban group. The mean age of COVID-19 patients was 30.55 years, notably younger than that of all patients.

**Table 3:** Top 15 health problems in the two hospitals in 2020 *The denominator of proportion is the total number of patients admitted to internal medicine wards in each hospital. COVID-19, coronavirus disease 2019.

Urban (Matsue)				Rural (Unnan)			
Rank	Health problem	Proportion*	ICD-10 code	Rank	Health problem	Proportion*	ICD-10 code
1	Congestive heart failure	6.9%	I500	1	Urinary tract infection	8.4%	N390
2	Pneumonitis due to inhalation of food and vomit	6.2%	J690	2	Polyp of colon	6.4%	K635
3	Urinary tract infection	5.9%	N390	3	Acute heart failure	4.1%	I509
4	Other forms of angina pectoris	3.7%	I208	4	Unspecified bacterial pneumonia	3.8%	J159
5	Pneumonia, unspecified organism	3.3%	J189	5	Pneumonitis due to inhalation of food and vomit	3.4%	J690
6	Cerebral infarction due to embolism of cerebral arteries	2.6%	I634	6	Cerebral infarction, unspecified	3.0%	I639
7	Dehydration	2.1%	E86	7	Pneumonia, unspecified organism	2.7%	J189
8	Persistent atrial fibrillation	2.1%	I481	8	Dehydration	2.2%	E86
9	Calculus of bile duct with cholangitis	2.1%	K803	9	Coma	2.2%	R402
10	Polyp of colon	1.6%	K635	10	Sepsis	2.2%	A419
11	Paroxysmal atrial fibrillation	1.6%	I480	11	COVID-19	1.8%	U071
12	Infectious gastroenteritis and colitis	1.4%	A090	12	Other peripheral vertigo	1.7%	H813
13	Parkinson’s disease	1.4%	G20	13	Cerebral infarction due to thrombosis of cerebral arteries	1.6%	I633
14	Supraventricular tachycardia	1.3%	I471	14	Congestive heart failure	1.4%	I500
15	Encounter for observation for other suspected diseases and conditions ruled out	1.1%	Z038	15	Syncope and collapse	1.2%	R55

SoP of urban and rural physicians

The result of the Mann-Whitney test, presented in Table 4, shows that the SoP-50% of the rural group was significantly higher than that of the urban group in 2018 (p = 0.0209). In 2020, there were 20 and 12 physicians in the urban and rural groups, respectively. In the urban group, the number of health problems addressed by a physician in a year was 1 as the lowest and 107 as the highest, and the median SoP-50% was 4.5, whereas, in the rural group, it was 8 as the lowest and 87 as the highest, and the median SoP-50% was 11. The result of the Mann-Whitney test indicated that the SoP-50% of the rural group was significantly higher than that of the urban group in 2020 (p = 0.0441).

**Table 4:** SoP-50% of urban and rural physicians Min, minimum; Max, maximum; IQR, interquartile range; SoP, scope of practice

Year	Group	Physicians (n)	Health problems (Min-Max)	SoP-50% Median, (IQR)	P-value
2018	Urban	18	(1–90)	6, (2–14)	0.0209
	Rural	7	(8–134)	16, (16–16)	
2020	Urban	20	(1–107)	4.5, (3–10.5)	0.0441
	Rural	12	(8–87)	11, (8.5–13)	

SoP of physicians pre and post-COVID-19 pandemic

We also analyzed the SoP of urban and rural physicians from the perspective of the pre and post-COVID-19 pandemic. The result of the Mann-Whitney test is shown in Table 5. No statistically significant differences were detected between 2018 and 2020 in the urban or rural group. In the urban group, the median SoP-50% was 6 in 2018 and 4.5 in 2020 (p=0.6915) while in the rural group, the median SoP-50% was 16 in 2018 and 11 in 2020 (p=0.2188).

**Table 5:** Changes in SoP-50% between pre-and post-COVID-19 SoP, scope of practice

Group	Year	SoP-50%, Median, (IQR)	P-value
Urban	2018	6, (2-14)	0.6915
	2020	4.5, (3-10.5)	
Rural	2018	16, (16-16)	0.2188
	2020	11, (8.5-13)	

## Discussion

Our results indicated that despite the same rural prefecture, similar patient background, and hospital capacity, physicians in rural districts have a significantly broader SoP as compared with those in the urban districts of Japan. Several previous studies have indicated the broad clinical comprehensiveness of rural doctors [12-15]; thus, the results of this study do not contradict these global trends but suggest that physicians working in Japanese rural community hospitals require a broader SoP as compared with their urban counterparts. There is a similar study conducted in Canada, which compared the geographic variation of SoP of rural and urban physicians [2]. The finding of our study is consistent with the above study, which revealed that rural physicians engage in a wider variety of clinical activities [2]. Therefore, it is globally common that rural physicians have a tendency to have a wider SoP. The findings of our study highlight that geographical variance in the SoP still occurs in the same rural prefecture.

The differences in the SoP of physicians in this study may be due to the geographically uneven distribution of specialists and the shortage of doctors in remote areas [16-17]. In general, doctors tend to be concentrated in urban areas [17]. However, the issue of the geographically uneven distribution of specialists in Japan is being discussed [18]. Our data analysis revealed specific issues. For instance, in the rural group, “Polyp of the colon” accounted for the largest proportion (5.7%) in 2018 and the second-largest proportion (6.4%) of all admitted patients in 2020, whereas it was only 1.5% in 2018 and 1.6% in 2020 in the urban group. This difference can be explained by the existence of specialized endoscopists in the urban region, where many colonoscopies are conducted in ambulatory settings. This medical procedure is more common in urban settings with more gastroenterologists and rectal surgeons [19]. Due to the absence of gastroenterologists in rural areas, most patients with "Polyp of the colon" are admitted for monitoring and care. Another notable finding is that “Atrial fibrillation” admissions constitute a relatively large proportion (5.7% in 2018 and 3.7% in 2020, persistent and paroxysmal combined) of all health problems in the urban group, whereas there were no admissions under this diagnosis in the rural group. This may be because there were arrhythmia specialists in the urban hospital who performed catheter ablation on those patients. The existence of cardiologists might have influenced a significant proportion of heart failure admissions in the urban group (5.5% vs. 4.5% in 2018 and 8.0% vs. 5.5% in 2020). These results suggest that organ-specific specialists might be unevenly distributed in the exact rural prefecture.

Our results also suggest the importance of medical education in infectious diseases. As illustrated in Tables 2-3, infectious diseases constitute a large proportion of health problems in the urban and rural groups, and their percentages were slightly higher in the rural group, both in 2018 and 2020 (21.4% vs. 16.1% in 2018 and 22.3% vs. 18.9% in 2020). This implies that clinical competency in infectious disease is crucial for a rural physician. However, there is a lack of systematic learning opportunities for infectious diseases in the Japanese undergraduate medical education curriculum [20]. One of the probable reasons is that infectious disease is a multiorgan field, and thus, it is not easy to design the curriculum in an educational system fragmented by organ specialties. Integration of clinical clerkship in rural community hospitals, wherein many patients are admitted for infectious diseases, may enrich medical education in this field.

This study also elucidated the possible issue of “care fragmentation” in urban community hospitals. Physicians whose SoP was extremely low and only addressed one category of health problem in the year were included in the urban group in this study. Meanwhile, no physician was dealing with solely one type of health problem in the rural group. This situation in the urban group could result in “care fragmentation,” which occurs when multiple healthcare providers from multiple subspecialties are involved in a patient's care [21]. This issue is critical, especially in an aging society where many patients have complicated multimorbidities, which can decrease their quality of life [22]. Japan has a free access system for medical care, which makes the issue of care fragmentation significantly more challenging to manage, as the system allows patients to visit any hospital they choose [23]. Consequently, the patients end up receiving medications from multiple health professionals, leading to “polypharmacy,” a common issue in older patients [24]. In an aging society, where many older people have various health problems, comprehensive care is crucial to developing a sustainable medical care system [23,25].

The findings of this study concerning the broad SoP of rural physicians can be helpful to physicians considering medical education and a medical career. The sustainability of rural healthcare systems has been challenged in many countries [26]. The rural training curriculum is one of the essential factors that attract physicians to rural locations [26]. Some medical universities in Japan are already implementing early intensive exposure to clinical clerkships in rural community hospitals as part of undergraduate programs for physician retention in remote areas [27]. Furthermore, Japan is implementing various strategies for rural healthcare maintenance such as providing scholarships in exchange for postgraduate rural services [27]. There are some successful examples of rural-oriented medical schools, such as Jichi Medical University in Japan, James Cook University in Queensland, Australia, and Memorial University in St. John’s, Canada [28-30]. Although these efforts contribute to the maintenance of rural healthcare to a certain degree, there is a need for additional incentives such as a scholarship. The best way for physician recruitment and retention is to emphasize the attractiveness of rural clinical practice [28]. Perhaps one might assume that rural physicians are under enormous stress because of the need to address a wide range of health problems with limited specialty consultation or advanced medical technology. Conversely, a study indicated that family physicians who provide a broader SoP reported significantly lower rates of burnout [29]. One of the systematic reviews on family physicians’ SoP revealed that those with diverse clinical activities have significantly higher job satisfaction [30]. In a recent study, it was further indicated that the defining factors for choosing rural practice include previous experience, preference for a rural lifestyle, desire for autonomy, a comprehensive SoP, and close relationships with patients [16]. Therefore, focusing on the SoP of rural physicians in the course of medical education could attract young doctors and medical students, as this could be an internal incentive for physician recruitment and retention.

Finally, we made an assessment regarding how the COVID-19 pandemic affected the clinical practice of the two hospitals. One of the notable changes was the number of admitted patients, which dropped from 2,142 to 1,776 in the urban group while it was relatively constant (1,075 to 1,023) in the rural group, even during the pandemic. This can be explained by the change in help-seeking behaviors of patients, based on the “stay-home” tendency and avoidance of unnecessary medical consultation. The lack of variation in the number of admitted patients in the rural group may be partly due to an increase in the number of physicians (from 7 to 12). However, another possible explanation is the increase in the proportion of patients who received comprehensive care in rural regions [10]. In addition, the early adaptation in the admission of COVID-19 patients was another possible reason for the similarity in the number of admitted patients in the rural group. The pandemic induced a decrease in medical care usage, and its influence is more evident in the urban than in the rural districts.

The influence of COVID-19 can also be found in the mean age of admitted patients. When comparing 2018 and 2020, there was a significant difference in the mean age of the admitted patients, both in the urban and rural groups. Notably, the mean age was higher in the urban group in 2020 than in 2018, whereas it was lower in 2020 than in 2018 in the rural group (Table 1). In other words, a contrast was observed in the age distribution in the two hospitals. This may be since COVID-19 patients, who were notably younger than other patients (mean age of 30.55 years), lowered the mean age of the admitted patients in the rural group in 2020. In the urban group, the increase in the mean age of patients during the pandemic may be due to decreased medical care usage, especially among younger patients.

Regarding the SoP of physicians, no significant difference was detected between pre or post-COVID-19 in the urban or rural group. This was contrary to our hypothesis that the rural group would show a wider SoP during the COVID-19 pandemic compared to pre-COVID-19 because of higher comprehensive care in the rural district during this period [10]. This may be since this study is limited to admitted patients, and including the outpatient population may result in a different outcome.

One of the limitations of this study is that only two hospitals in rural Japanese settings were considered. To elucidate the geographical variation in physicians’ SoP in Japan and across the world, future studies should include more hospitals across multiple prefectures and other countries. As Japan is one of the leading countries in terms of aging societies, these findings can be applied to countries preparing for aging issues and medical education in aging societies. Furthermore, this study, being of a cross-sectional design, cannot reveal the cause-and-effect relationship between SoP and geographical differences. Future research should investigate this relationship by conducting longitudinal studies. Regarding the influence of COVID-19, future studies may be needed to include the outpatient population to identify its effect with evidence.

## Conclusions

Rural Japanese physicians in rural districts have a wider SoP than in urban districts. Our study has revealed that this seemingly well-known tendency remained even in the same rural prefecture of Japan. In addition, this phenomenon remained unchanged even during the COVID-19 pandemic, which has brought many changes in the way medical care is provided.

This study also demonstrates the importance of medical education when handling infectious diseases, which constitute a large part of the health problems in community hospitals in Japan. The integration of clinical clerkship in rural hospitals may enrich medical education in infectious diseases. The findings of this study can be utilized in policy-making for the recruitment and retention of physicians in rural hospitals.
